# Regional White Matter Volumes Correlate with Delay Discounting

**DOI:** 10.1371/journal.pone.0032595

**Published:** 2012-02-29

**Authors:** Rongjun Yu

**Affiliations:** 1 Department of Psychology and Center for Studies of Psychological Application, South China Normal University, GuangZhou, GuangDong, China; 2 MRC Cognition and Brain Sciences Unit, University of Cambridge, Cambridge, United Kingdom; University of Maryland, College Park, United States of America

## Abstract

A preference for immediate gratification is a central feature in addictive processes. However, the neural structures underlying reward delay tolerance are still unclear. Healthy participants (n = 121) completed a delay discounting questionnaire assessing the extent to which they prefer smaller immediate rewards to larger delayed reward after undergoing magnetic resonance imaging (MRI) scanning. Whole brain voxel-based morphometric analysis shows that delay discounting severity was negatively correlated with right prefrontal subgyral white matter volume and positively correlated with white matter volume in parahippocampus/hippocampus, after whole brain correction. This study might better our understanding of the neural basis of impulsivity and addiction.

## Introduction

People prefer to obtain reward immediately rather than in future. Such preference has been vividly demonstrated in the delay of gratification paradigm commonly used in developmental and personality psychology. In the famous ‘marshmallow test’, which measures how long a child can resist eating a small immediate reward in order to get a larger reward some time later, it has been shown that 4-year-old children who delayed gratification longer developed into more cognitively and socially competent adolescents, achieving higher scholastic performance and coping better with frustration and stress [1]. The interpretation of delaying gratification focuses on individuals' willpower to resist the temptations of the immediate reward. Although not the equivalent measures [2], delay discounting procedures are used in behavioural economics and nonhuman animal behavioural studies to precisely assess delay-related impulsive behaviour. Delay discounting refers to that phenomenon that the present value of a future reward reduces as the delay to that reward increases. Delay discount rates are relatively stable over time periods of more than 1 year [3], [4]. They correlate negatively with college grade point average [5], as well as adolescent academic performance [6]. A number of studies have found steeper discounting functions among various substance abusers, such as alcoholics, drug addicts, or heavy smokers, than among those of matched control groups [7], [8], [9], [10], [11], [12], [13], [14], [15]. Although making choices in favor of long-term goals is important for both individuals and our society, the underlying neural correlates of delay related impulsive behaviours in humans are still not clear.

Since the delay of gratification paradigm (e.g. Stanford marshmallow experiment) only provides qualitative measure of impulsivity, few neuroimaging studies have been conducted using this paradigm. In a recent longitudinal study using functional magnetic resonance imaging (fMRI), low delayers, those who have low resistance to temptation, as measured originally by the delay of gratification task 40 years ago, showed lesser recruitment of inferior frontal gyrus and greater recruitment of the ventral striatum when resisting alluring cues [16], suggesting that resisting temptation is supported by frontostriatal circuitries.

A large number of human neuroimaging studies have used the delay discounting paradigm to investigate the neural associates of delay discounting choices but the findings are inconsistent. Some studies found specific brain regions, such as ventral striatum, medial orbitofrontal cortex, medial prefrontal cortex, posterior cingulate cortex, and left posterior hippocampus, are preferentially activated by decisions involving immediately available rewards [17], [18], [19]. However, another study found that brain regions, including the ventral striatum, medial prefrontal cortex and posterior cingulate cortex are engaged in evaluating both delay reward and immediate reward [20]. Lesion studies in humans also found mixed results. One study reported that delay discounting is not affected by lesion to the ventromedial prefrontal cortex [21], whereas another study found that damage to medial orbitofrontal cortex increased significantly the preference for small-immediate over larger-delayed rewards [22]. These studies raise questions on whether there are some specific brain regions dedicated to delay discounting processing or delay discounting is supported by a general brain network, e.g. brain regions that support cognitive calculation and general decision making. One approach to answer this question is to correlate individual differences in delay discounting with brain structure characteristics.

To date, only one preliminary study has investigated the correlation between delay discounting rates and prefrontal grey matter volume in a small group (n = 29) using a region of interest (ROI) approach [23]. Here, voxel-based morphometry (VBM) is used to examine the associations between regional white matter (WM) and gray matter (GM) volumes and individual differences in delay discounting. Compared to the ROI approach, which manually delineate GM/WM volumes in pre-specified regions only, VBM allows for examining the entire brain on a voxel-by-voxel basis in a fully automated manner, without having to specify in advance regions of interest. Delay discounting was chosen because it provides a precise quantitative measurement of delay-based impulsive behaviours. Previous neuroimaging studies in humans and animal studies have identified candidate brain regions in which activity was associated with delay discounting, especially the prefrontal and temporal regions [24], [25]. I hypothesized that the local structure of these regions might reflect an individual's ability to tolerate reward delay.

## Methods

### Participants

One hundred and twenty one healthy right-handed volunteers (58 male, mean age and SD 25.27±5.16, ranging from 18 to 49) participated in the experiment. Participants were recruited from the community through advertisements. All subjects were screened for psychiatric and nonpsychiatric medical disorders using the Structured Clinical Interview for the Diagnostic and Statistical Manual of Mental Disorders, 4th edition (DSM-IV; SCID). All subjects were right-handed, had no history of any neurological or psychiatric disorder, had no drug dependence, and were not currently taking any medications. Participants were compensated for their participation with 20 pounds. All recruited participants were informed about all procedures of the experiment and written informed consents were obtained. This study was approved by the Cambridge Research Ethics Committee review board.

### Questionnaire

Outside scanner, participants were asked to fill in the delay discounting questionnaire, which has good to excellent internal consistency [6]. There are 27 choice trials in the questionnaire. Each trial consists of one smaller, immediate reward and one larger, delayed reward (i.e., “Would you prefer £54 today, or £55 in 117 days?”). Participants indicate which alternative they would prefer to receive by circling it. The behavioural choices were fitted to the hyperbolic function: V = A/(1+kD), where V is the value of the delayed outcome (i.e., the indifference value), A is the fixed delayed reward, D is the length of the delay, and k expresses the steepness of the discount function. For example, one offered participants a choice between ‘£33 today’ and ‘£80 in 14 days’. A participant with a discount rate of 0.10 would be indifferent between these two rewards. Therefore, if a participant chose the immediate reward on this trial, then one could infer that this person has a discount rate greater than 0.10. Another question offered participants a choice between ‘£31 today’ and ‘£85 in 7 days’. A participant with a discount rate of 0.25 would be indifferent between these two rewards. Therefore, if the same participant chose the delayed reward on this trial, then one could infer that this person has a discount rate less than 0.25. The two trials together imply that this person has a discount rate between 0.10 and 0.25. We used the geometric mean of this interval as our estimate of the person's k value. In the example, this yields k = 0.16. The participant was assigned to the value that yielded the highest consistency among his or her choices. Finally, to obtain a single rate estimate for each participant, we computed the geometric mean of the three rates for the small, medium and large reward magnitudes [15]. Higher values of k indicate that the delayed rewards are being discounted more steeply, meaning that the subject is more impulsive. Because raw k values were skewed toward low values and were not normally distributed, the discounting parameter k was normalized by logarithmic transformation (hereafter *lnK*) for subsequent analysis. Individuals with consistency lower than 75% were excluded in the following analysis.

### Scanning Procedure

A high-resolution structural magnetization prepared rapid gradient echo scan (voxel size = 1.3×1.3×1 mm, repetition time = 2250 ms, echo time = 2.99 ms, inversion time = 900 ms, flip angle = 9°, total scan time = 4 min 16 s) was acquired on a Siemens 3-T MRI scanner for all participants.

### Data Analysis

VBM analysis was performed in SPM5 (Welcome Trust Centre for Neuroimaging, London, UK), which enables automated spatial normalization, tissue classification, and radiofrequency bias correction to be combined within the segmentation step. The segmented images were smoothed using a Gaussian kernel of 8-mm full-width at half-maximum (FWHM). An absolute threshold mask was set at 0.1 to ensure that the voxels included in the analysis had a higher probability of being WM or GM, respectively. Global WM/GM volume was included as a covariate of no interest in the analysis of WM/GM volume differences, to account for any gross differences in total WM/GM volume across participants [26]. For example, the correlation between WM and *Ink* was controlled for global WM (and not GM) and the converse was done for the correlation between GM and *Ink*. Age and gender were also included as covariates of no interest in all models [27]. A false discovery rate (FDR) corrected threshold of P<0.05 at cluster level was set, corresponding to voxel-wise P<0.001, cluster size = 162, shared edge.

## Results

The *lnK* was −4.80±SD 1.40, ranging from −8.29 to −1.84. There was no gender difference, p>0.3. The *lnK* was not associated with age, global WM volume, or global GM volume (p values >0.05). Similar to previous studies, the consistency of choices are very high [15], [28]. Mean consistencies were 98%±4%, 98%±4%, and 97%±5%, for the small, medium, and large delayed reward conditions, respectively. The lowest consistency was 78%. The high consistency in participants' choices suggests that they were generally very careful in making their choices, even though rewards were hypothetical.

**Figure 1:**
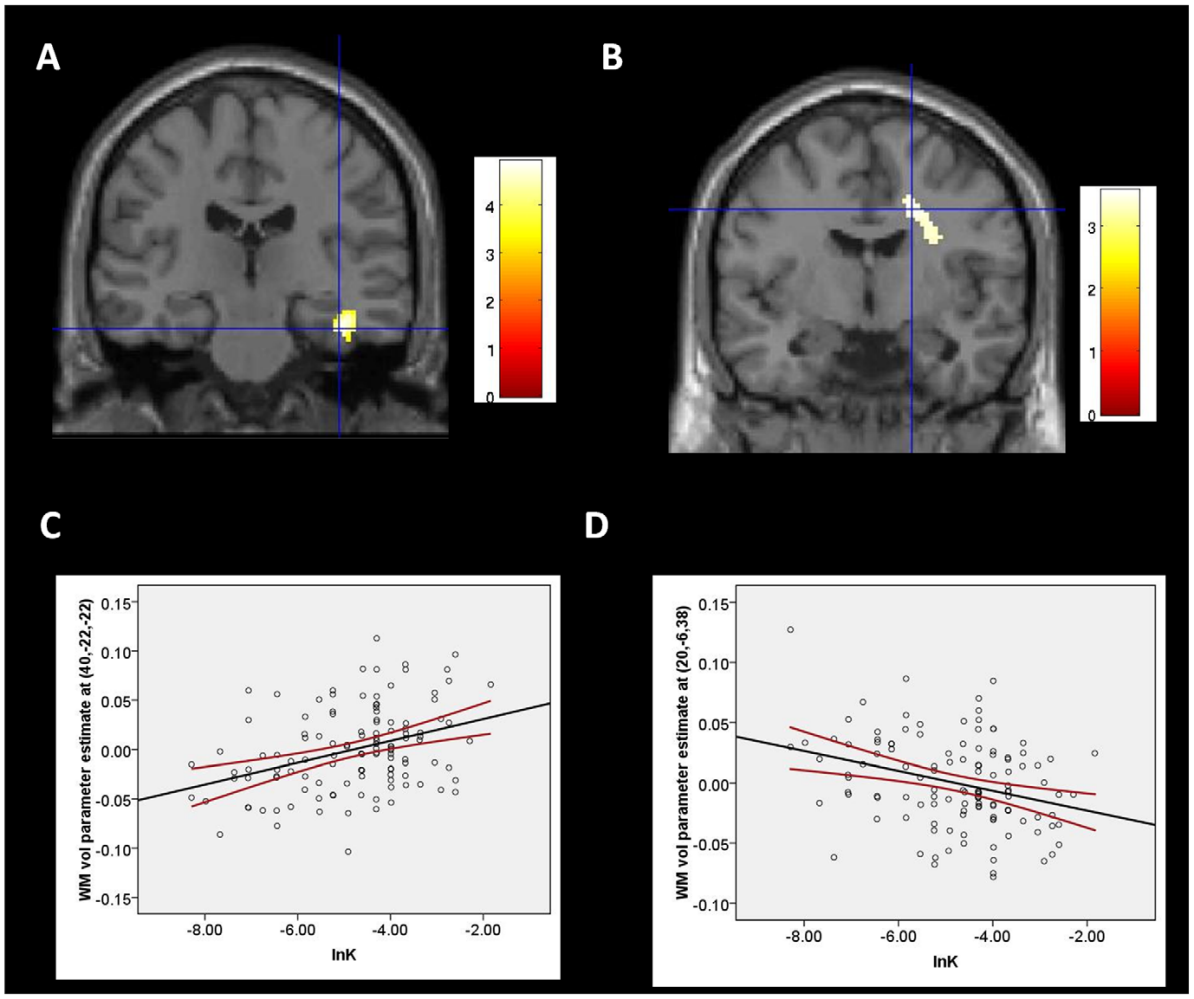
Regional white matter volume correlated with discounting severity. White matter volume in the parahippocampus/hippocampus (A) and right prefrontal subgyral area (B) correlated with delay discounting rates after whole brain correction. Scatterplot shows the size of white matter volume changes in the parahippocampus/hippocampus (C) and the right prefrontal subgyral area (D) at the peak voxel (a voxel with local maxima) as a function of delay discounting rates (i.e. the *Ink*). For display purposes, maps are thresholded at p<0.001, uncorrected.

The whole brain analysis revealed that WM in the right parahippocampus extending to the right hippocampus [40,−22,−22, peak z = 4.57, cluster size = 365 voxels, cluster level corrected P^FDR^<0.005] showed a positively correlation with the *lnK*, whereas WM volumes of the right prefrontal subgyral area [20,−6,38, peak z = 3.46, cluster size = 162 voxels, cluster level corrected P^FDR^<0.05] showed a negative correlation with the *lnK* (see Figure 1). Prefrontal cluster still significantly correlated with discounting rates even after the data point that may be an influential outlier was removed from the analysis. These results are whole brain corrected and controlled for age, gender and absolute whole brain WM volumes. No significant correlation between regional GM volume and the *InK* was found after whole brain correction. For the completeness' sake, results were also shown at P<0.001 with a 10 voxel extent threshold, uncorrected (see Table 1).

**Table 1:** Regional white matter (WM) and grey matter (GM) volume showing significant correlations with discounting severity (i.e. the *Ink*) at P<0.001 with a 10 voxel extent threshold, uncorrected.

Brain Regions	Z scores	Voxels	MNI Coordinates		
			X	Y	Z
Positive correlation with discounting severity (WM)					
R Parahippocampus	4.68	365	40	−22	−24
L Parahippocampus	4.32	55	−38	−22	−24
R Middle Temporal Cortex	4.46	47	58	−40	−14
L Middle Temporal Gyrus	4.01	15	−58	−40	−14
L Ventral Anterior Cingulate Cortex	3.5	37	−6	34	−14
L Inferior Frontal Cortex	3.41	16	−30	24	−10
Negative correlation with discounting severity (WM)					
R Prefrontal Subgyral Area	3.46	162	48	9	6
R Dorsal Anterior Cingulate Cortex	3.46	16	12	30	30
Positive correlation with discounting severity (GM)					
R Inferior Temporal_Cortex	3.37	42	48	−14	−38
Negative correlation with discounting severity (GM)					
L Angular Gyrus	4.03	42	−36	−56	34
R Middle Temporal Gyrus	3.86	37	54	−38	−12
R Parahippocampa Gyrus	3.5	34	44	−20	−18
L Lingual Gyrus	3.47	42	−20	−72	−6

These results are shown for the completeness sake and need to be interpreted with caution.

## Discussion

In this VBM study, I found that individuals who have steeper delay discounting rates (more impulsive) have smaller WM volumes in the prefrontal cortex. They also have relatively larger WM volumes in the right parahippocampus and hippocampus.

The prefrontal cortex has been implicated in regulating cognitive and emotional processes [29]. A recent diffusion tensor imaging (DTI) study showed that WM integrity in the right frontal region cluster and in the left temporal lobe cluster was negatively correlated with discounting rates [30]. Another morphometric study (n = 29) using region of interest (ROI) analysis has revealed a negative partial correlation between *lnK* and the GM volume in the lateral frontal cortex [23]. Our finding of reduced WM volume in the right prefrontal subgyral area converges with these results and suggests that the cognitive control dysfunction may lead to the inability to resist the temptation of immediate reward [29].

It is interesting to note that WM volumes in the parahippocampus/hippocampus were positively correlated with discounting rates. Lesion of hippocampus in rats leads to high impulsive behaviours, such as more likely to choose small but immediate reward [24]. Neuroimaging studies in humans have shown that the parahippocampus/hippocampus plays an important role in self-projection into the future [31]. Increased functional coupling between ACC and hippocampus was associated with increasing shifts toward more future-minded choice behaviour [32]. The parahippocampal gyri exhibited enhanced activity for long versus short delay, suggesting that during intertemporal choice, decision makers simulate the impending delay via a process of prospection [33]. Patients with hippocampal amnesia also cannot imagine new experiences [34]. It is possible that an individual's discounting of future events depends on his or her ability to remember past events. Consideration and valuation of the future events has been shown to overlap with processes associated with memory or valuation of the past [35]. Discounting of future rewards are correlated with discounting of past rewards [36]. A recent study demonstrated that rates of discounting of delayed rewards were significantly reduced among those who received memory training but were unchanged among those who received control training [37]. An interesting hypothesis for future studies to test is that hippocampal human patients would show abnormal temporal discounting. Together with the present work, these findings emphasize an important role for parahippocampus/hippocampus in delay discounting. Because the functional significance of larger white matter volumes is still not clear, the directionality of the correlation should be interpreted with caution. It is worth mentioning that size does not always correlate positively with performance. Larger regional white matter volumes are not always beneficial and they do not necessarily suggest better functions associated with those regions. For example, previous studies found that larger white matter volume was positively associated with number of intrusions [38]. Further research is needed to systematically relate white matter volume to memory ability before any firm conclusions can be made.

The basal ganglia also play a crucial in reward processing. Specially, ventral striatum has been found to mediate the delay discounting in several fMRI studies [39], [40]. Individuals who discounted future reward more steeply, as measured outside the scanner, exhibited more recruitment in the ventral striatum in response to both gains and losses [39]. Caudate lesion rats showed abnormal preference to immediate reward [41]. It is surprising that no significant difference in WM or GM among these regions was found in the present study. One possibility is that dynamic BOLD responses to immediate rewards at the individual level are highly variable and may highly depend on the experimental contexts [17], [20], [42], [43]. Whether there is a structural correlation in this region with delay discounting or not waits for future investigation.

There are some limitations of this study which should be considered in interpreting our results. First, I used hypothetical rather than real rewards. Previous studies suggest that hypothetical and real rewards are discounted similarly [9], [44], [45], [46]. Nevertheless, future studies are needed to replicate the current investigation using real rewards. Second, one may argue that the monetary delay discounting task used was quite short and may not provide the most precise estimate of discount rates. Although the present version of task has been used extensively in previous studies [15], [47], [48], [49], future studies may use a longer task to replicate the current findings. Third, the IQ scores were not measured in this study. IQ is associated with delay discounting [50], [51], as well as with brain structural features including grey matter density and thickness [52], [53]. Other information regarding demographic characteristics, such as years of education [54], ethnic background [55], was not collected. These variables may contribute to the effects observed in the current study. Future studies should carefully control for these variables and examine their influence on the brain structures. Finally, our sample may not be representative of the general population, since individuals with a history of any psychiatric disorder and individuals taking any medications were excluded. However, a large number of studies have demonstrated that psychiatric disorders and medications modulate brain structures [56]. Using a healthy sample allows controlling for confounding factors such as psychiatric disorders and medications.

In conclusion, this study provides further evidence that individual differences in delay discounting are associated with the volume of specific brain structures. It highlights the possibility that our ability to tolerant reward delay may be associated with brain regions implicated in executive control and mental simulation.
